# Supplementary material for: An anti-influenza combined therapy assessed by single cell RNA-sequencing
Source: Commun Biol. 2022 Oct 10;5:1075. doi: 10.1038/s42003-022-04013-4 (PMC9549038; doi:10.1038/s42003-022-04013-4)
Supplement: Supplementary file 2 — Supplementary Information [file 42003_2022_4013_MOESM2_ESM.pdf]

Supporting Information

An anti-influenza combined therapy assessed by single cell RNA-sequencing

Chiara Medaglia[§*1], Ilya Kolpakov[§2], Arnaud Charles-Antoine Zwygart[1], Yong Zhu[3], Samuel Constant[4], Song Huang[4], , Valeria Cagno[5], Emmanouil T. Dermitzakis[2], Francesco Stellacci[3], Ioannis Xenarios[2] and Caroline Tapparel*[1].

[1] Department of Microbiology and Molecular Medicine, University of Geneva, Geneva 1206 Switzerland.
[2] Health 2030 Genome Center, Geneva 1202, Switzerland.
[3] Insitute of Materials, Ecole Polytechnique Fédérale de Lausanne, Lausanne 1015, Switzerland.
[4]Epithelix Sas, Geneva 1228, Switzerland.
[5]Faculty of Biology and Medicine, Université de Lausanne, Lausanne 1011, Switzerland.

§ These authors contributed equally.
*corresponding authors: chiara.medaglia@unige.ch, caroline.tapparel@unige.ch.

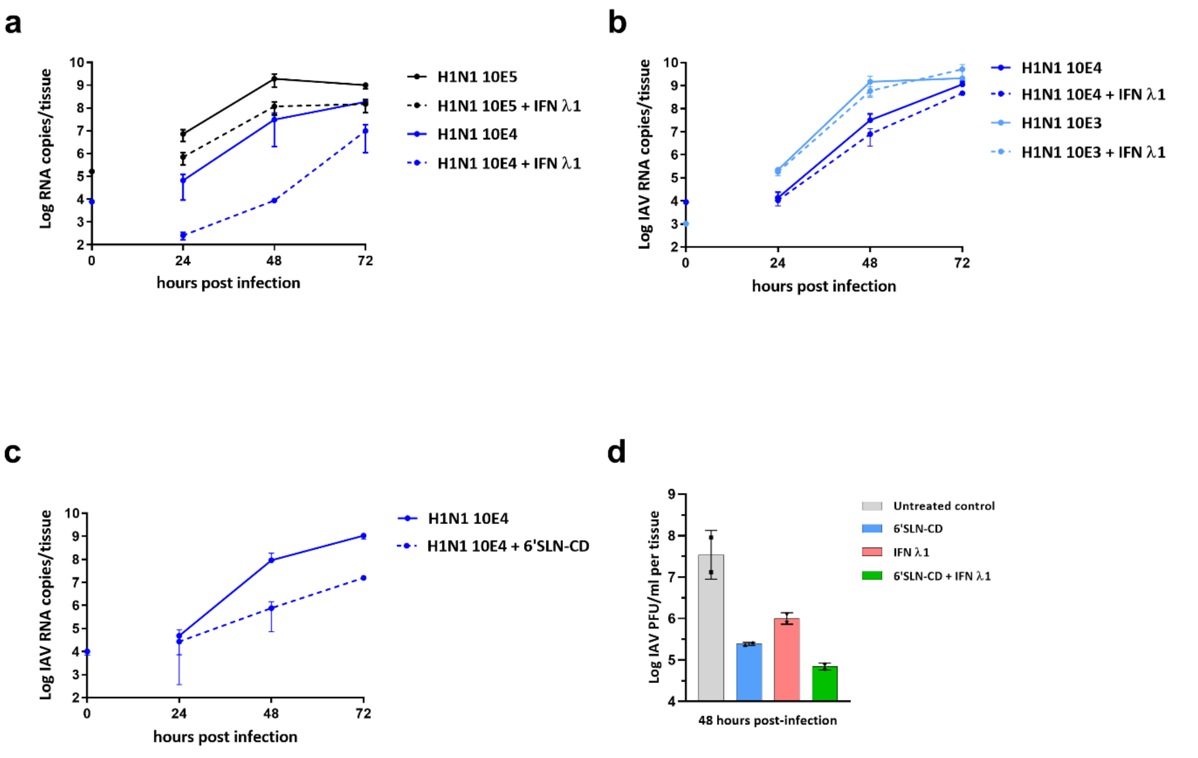

Supplementary Figure 1. Antiviral activity of IFN λ1 and 6'SLN-CD in HAE. HAE developed from a single donor were treated on their basal side with IFN λ1 (5.5 ng/insert) either starting at 24 hbi **a)**, or at 8 hpi **b)**, and infected with different numbers of RNA copies of clinical A/Switzerland/3076/2016 H1N1 (0 h corresponds to the time of viral inoculation). IFN λ1 was then administered daily up to 48 hpi. **c)** 6'SLN-CDs (60 µg/insert) were administered daily on the apical side of the tissues, starting from 8 hpi and up to 48 hpi. Viral replication was assessed measuring the apical release of IAV by RT-qPCR. **d)** Bar plot showing IAV replication in HAE treated with 6'SLN-CD only, or with IFN λ1 only, or with both compounds according to Figure 1b. Viral replication was assessed measuring the apical release of IAV by plaque assay, performed in MDCK cells. The results were obtained using HAE developed from

different donors and represent the mean and standard deviation from two independent experiments. HAE = human airway epithelia; hbi = hours before infection; hpi = hours after infection.

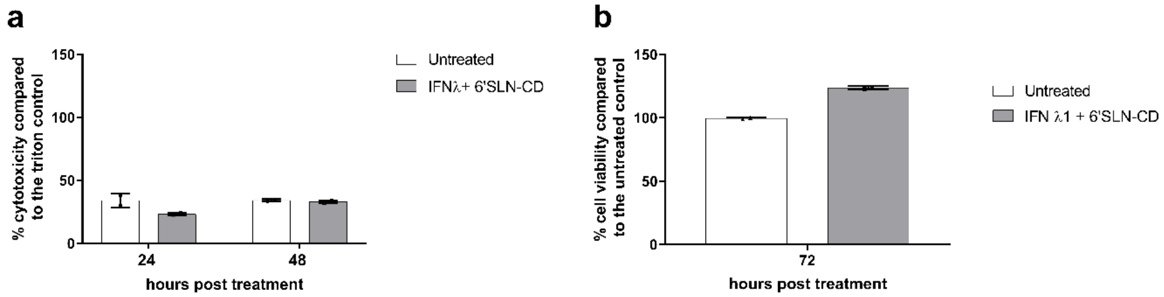

Supplementary Figure 2. Toxicity assessment of IFN λ1 + 6'SLN-CD in uninfected HAE. a) Measurement of cellular cytotoxicity by LDH assay. The percentage of LDH release was calculated compared to the triton cytotoxicity control. **b)** Measurement of cell metabolic activity by MTT assay. The percentage of MTT reduction into formazan was calculated relatively to the untreated control. The tissues were treated daily with 6'SLN-CD on their apical surface and with IFN λ on their basal side (60 µg and 5.5 ng per tissue, respectively, in PBS), for 72 h. The MTT assay was performed at 72 hours post treatment (hpt), while the LDH assay was performed at both 24 and 48 hpt on the apical sides of the tissues. Untreated control tissues (untreated) and cytotoxicity control tissues were treated on their apical side with PSB or with PBS-Triton 5%, respectively. The results represent the mean and standard deviation from two independent experiments. Paired sample t-test analysis showed no significant difference between untreated and treated conditions for both the LDH assay ($p = 0.43$) and the MTT assay ($p = 0.06$). HAE = human airway epithelia.

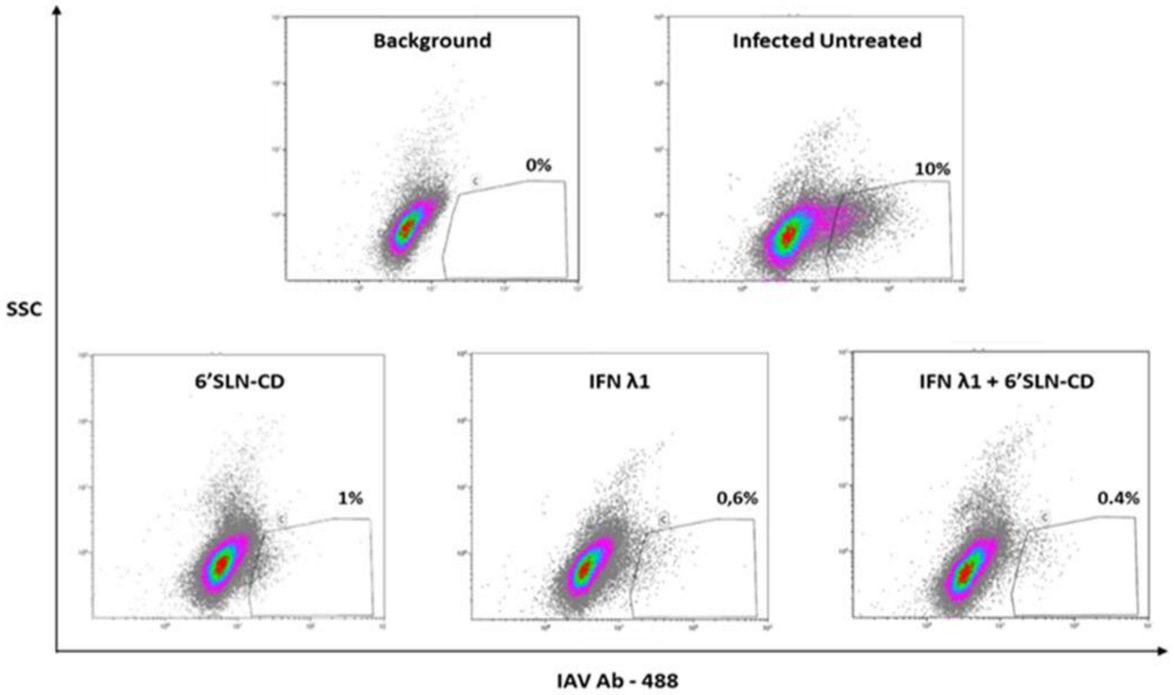

Supplementary Figure 3. Flow cytometry (FACS) analysis of infected HAE cells. The results were obtained from the same tissues shown in figure 1b and are representative of two

independent experiments. HAE developed from a pool of donors were infected with 10^3 RNA copies of clinical A/Switzerland/3076/2016 H1N1 and treated or not with 6’SLN-CDs (60 µg/tissue, administered daily starting at 8 hpi), or with IFN λ (5,5 ng/tissue, administered daily starting from 24h before infection), or with both compounds. At 48 hpi the tissues underwent enzymatic digestion and staining with an antibody (Ab) targeting IAV nucleoprotein. The gating was defined based on the background signal obtained from an infected tissue stained with the 2nd Ab alone. HAE = human airway epithelia.

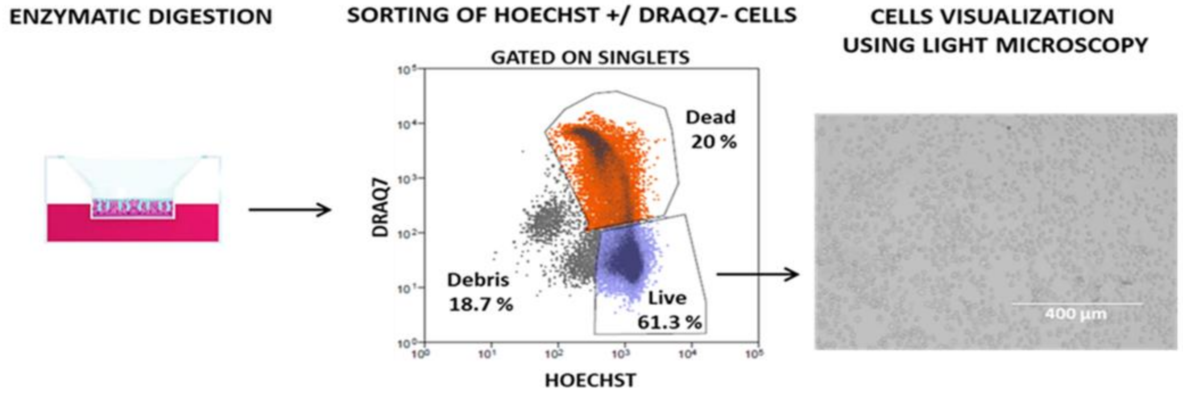

Supplementary Figure 4. HAE dissociation protocol for scRNA sequencing. Upon enzymatic digestion, cells were stained with Hoechst to label the nuclei and with DRAQ7 to exclude non-viable cells. Hoechst +/DRAQ7 – cells were sorted, visualized at the light microscope and then processed for scRNA-seq analysis. HAE = human airway epithelia.

a

Hallmark genes	Cell type
TP63	Basal
ITGA6	
KRT5	
KRT17	
LY6D	
KRT4	Secretory
KRT8	
CYP2F1	
KRT7	
VMO1	
SPRR3	
AGR2	
BPIFB1	
BPIFA1	
SCGB1A1	
SCGB3A1	
LYZ	
MUC5AC	
FOXJ1	Ciliated
TPPP3	
STOLM3	
ERICH3	
LRRC23	
PIFO	
IFT57	

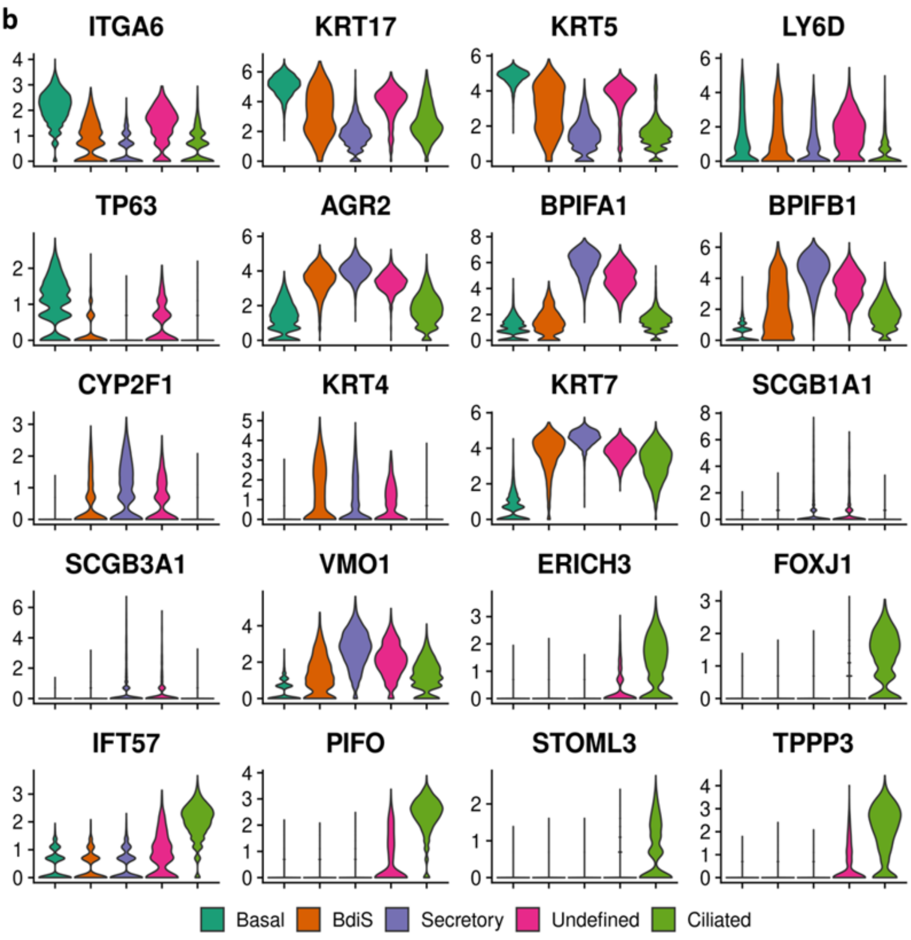

Supplementary Figure 5. a) Hallmark genes used to annotate the main human epithelial respiratory cell types. **b)** Violin plots showing the expression distribution of cell-type specific hallmark genes across

the HAE cell clusters described in Figure 2D. HAE = human airway epithelia.

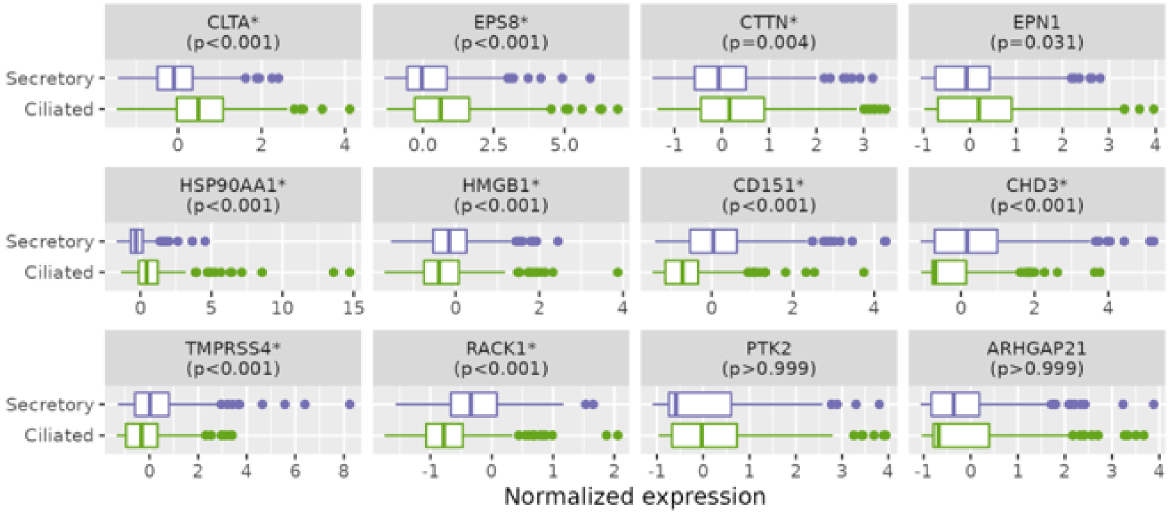

Supplementary Figure 6. Expression of host factors involved in IV replication across secretory and ciliated cells in steady state conditions. The figure shows the box-and-whisker plots of normalized gene expression. The x scales are Pearson residuals from SCTransform binomial regression model, while p-values are from Mann-Whitney-Wilcoxon test with multiple testing adjustment (see Methods).

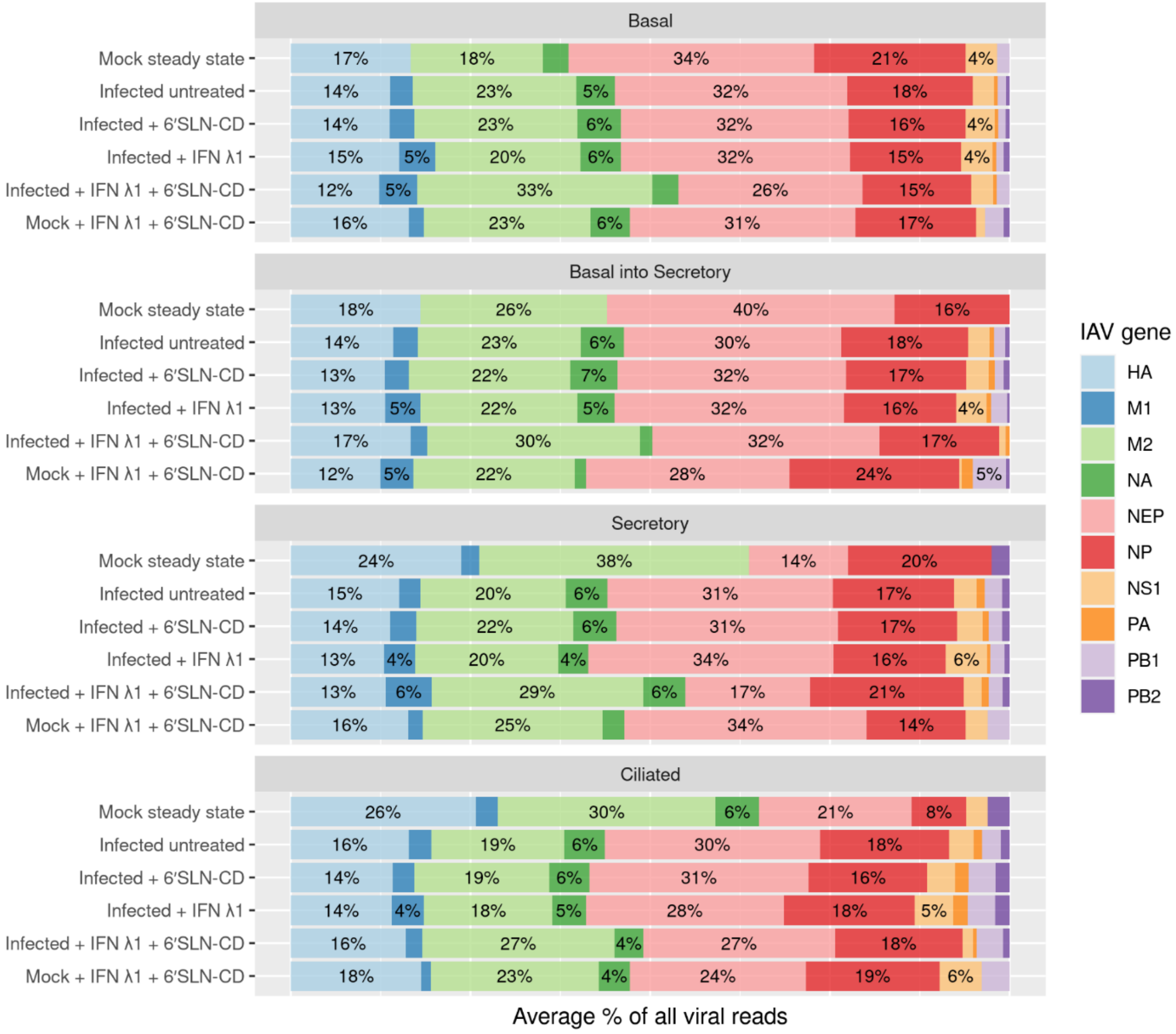

Supplementary Figure 7. Prevalence of IAV genes in IAV transcripts. The figure shows average within-cell fractions of specific IAV transcripts in all IAV transcripts (across cells having at least one IAV transcript).

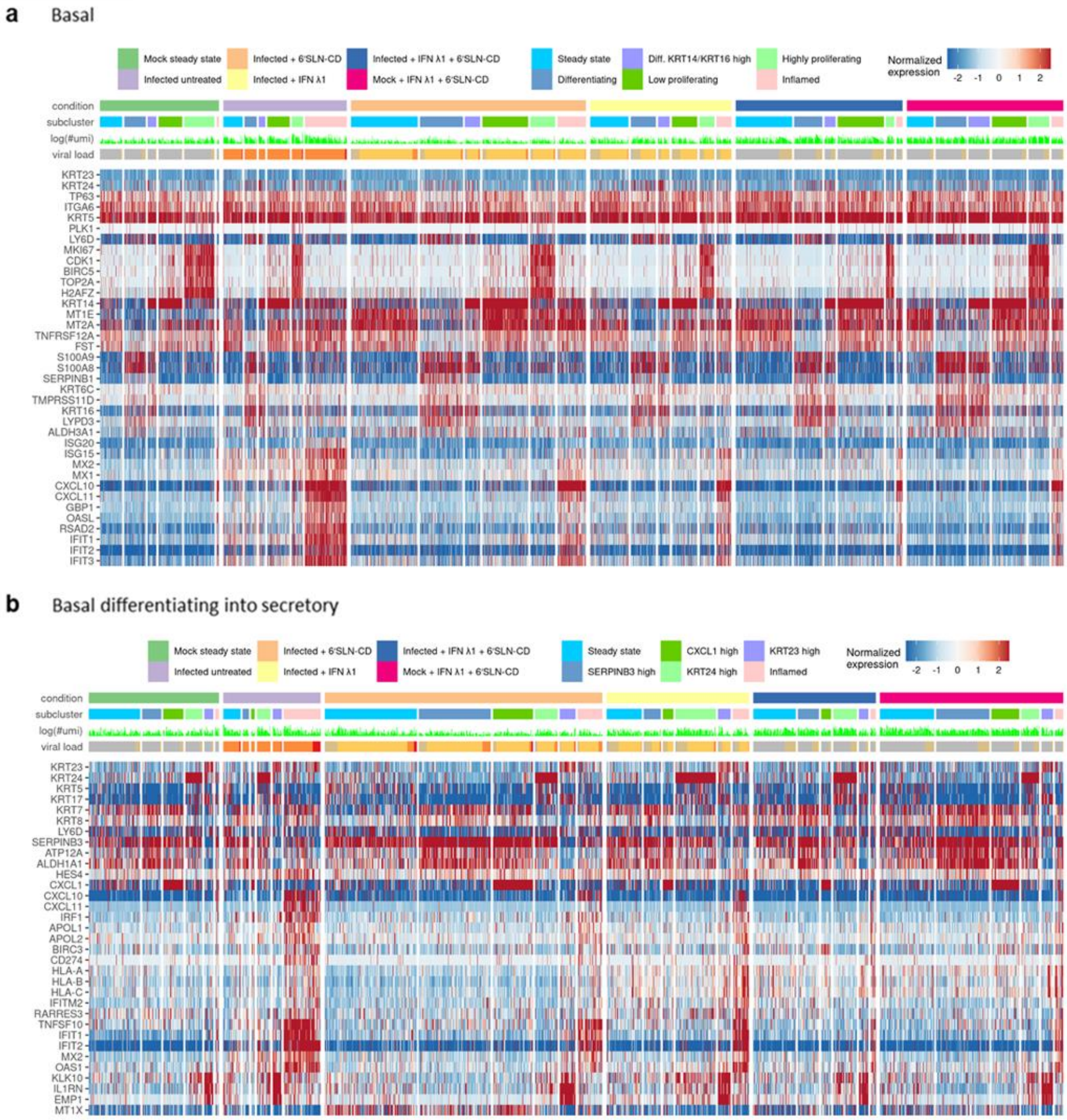
a Basal
Mock steady state
Infected untreated
Infected + 6'SLN-CD
Infected + IFN λ1
Infected + IFN λ1 + 6'SLN-CD
Mock + IFN λ1 + 6'SLN-CD
Steady state
Differentiating
Diff. KRT14/KRT16 high
Low proliferating
Highly proliferating
Inflamed
Normalized expression
-2 -1 0 1 2
condition
subcluster
log(#umi)
viral load
KRT23
KRT24
TP63
ITGA6
KRT5
PLK1
LY6D
MKI67
CDK1
BIRC5
TOP2A
H2AFZ
KRT14
MT1E
MT2A
TNFRSF12A
FST
S100A9
S100A8
SERPINB1
KRT6C
TMPRSS11D
KRT16
LYPD3
ALDH3A1
ISG20
ISG15
MX2
MX1
CXCL10
CXCL11
GBP1
OASL
RSAD2
IFIT1
IFIT2
IFIT3
b Basal differentiating into secretory
Mock steady state
Infected untreated
Infected + 6'SLN-CD
Infected + IFN λ1
Infected + IFN λ1 + 6'SLN-CD
Mock + IFN λ1 + 6'SLN-CD
Steady state
SERPINB3 high
CXCL1 high
KRT24 high
KRT23 high
Inflamed
Normalized expression
-2 -1 0 1 2
condition
subcluster
log(#umi)
viral load
KRT23
KRT24
KRT5
KRT17
KRT7
KRT8
LY6D
SERPINB3
ATP12A
ALDH1A1
HES4
CXCL1
CXCL10
CXCL11
IRF1
APOL1
APOL2
BIRC3
CD274
HLA-A
HLA-B
HLA-C
IFITM2
RARRES3
TNFSF10
IFIT1
IFIT2
MX2
OAS1
KLK10
IL1RN
EMP1
MT1X

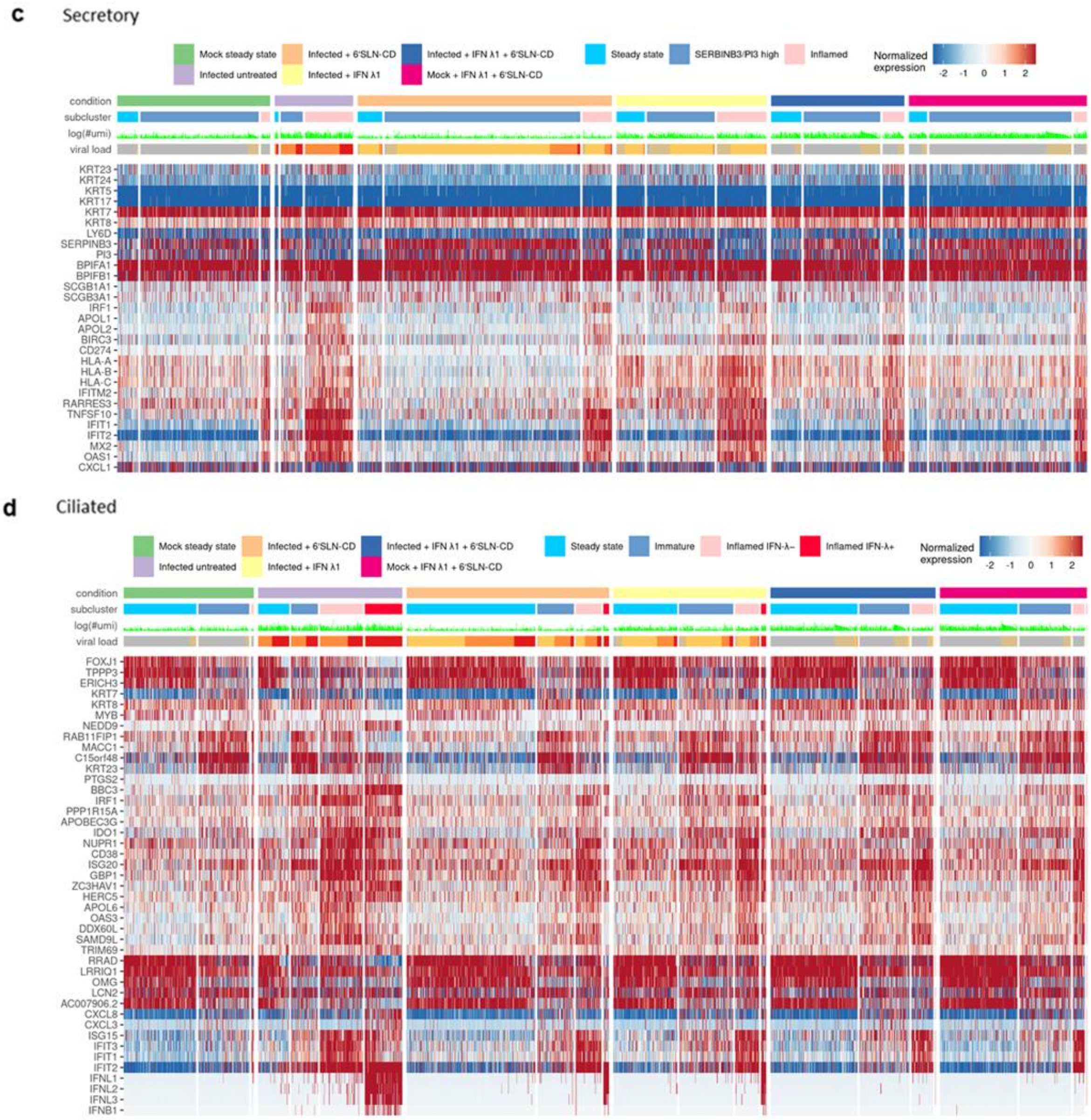

Supplementary Figure 8. Gene expression profiles of identified HAE cell types and their subclusters across experimental conditions. a) basal cells, **b)** basal cells differentiating into secretory cells, **c)** secretory cells, and **d)** ciliated cells. The values visualized on the heatmaps are Pearson residuals from SCTransform binomial regression model fitted to UMI counts (*70*) (see Methods). Conditions as in Figure 2c and viral load categories as in Figure 3a. Per-cell UMI counts visualized in light green (on log scale) include viral transcripts, represented with the same color code of figure 3b. HAE = human airway epithelia.
