# Supplementary material for: An anti-influenza combined therapy assessed by single cell RNA-sequencing
Source: Commun Biol. 2022 Oct 10;5:1075. doi: 10.1038/s42003-022-04013-4 (PMC9549038; doi:10.1038/s42003-022-04013-4)
Supplement: Supplementary file 3 — Reporting summary [file 42003_2022_4013_MOESM3_ESM.pdf]

Corresponding author(s): DBPR COMMSBIO-22-0469-T

Last updated by author(s): 22.02.2022

Reporting Summary

Nature Portfolio wishes to improve the reproducibility of the work that we publish. This form provides structure for consistency and transparency in reporting. For further information on Nature Portfolio policies, see our Editorial Policies and the Editorial Policy Checklist.

Statistics

For all statistical analyses, confirm that the following items are present in the figure legend, table legend, main text, or Methods section.

n/a	Confirmed	
☐	☒	The exact sample size (*n*) for each experimental group/condition, given as a discrete number and unit of measurement
☐	☒	A statement on whether measurements were taken from distinct samples or whether the same sample was measured repeatedly
☐	☒	The statistical test(s) used AND whether they are one- or two-sided<br>*Only common tests should be described solely by name; describe more complex techniques in the Methods section.*
☐	☒	A description of all covariates tested
☐	☒	A description of any assumptions or corrections, such as tests of normality and adjustment for multiple comparisons
☐	☒	A full description of the statistical parameters including central tendency (e.g. means) or other basic estimates (e.g. regression coefficient) AND variation (e.g. standard deviation) or associated estimates of uncertainty (e.g. confidence intervals)
☐	☒	For null hypothesis testing, the test statistic (e.g. *F*, *t*, *r*) with confidence intervals, effect sizes, degrees of freedom and *P* value noted<br>*Give P values as exact values whenever suitable.*
☒	☐	For Bayesian analysis, information on the choice of priors and Markov chain Monte Carlo settings
☒	☐	For hierarchical and complex designs, identification of the appropriate level for tests and full reporting of outcomes
☐	☒	Estimates of effect sizes (e.g. Cohen's *d*, Pearson's *r*), indicating how they were calculated

Our web collection on statistics for biologists contains articles on many of the points above.

Software and code

Policy information about availability of computer code

Data collection	No software was used
Data analysis	The code for the analysis in the paper is available at GitHub repository https://github.com/ilya-kolpakov/anaic-analysis/

For manuscripts utilizing custom algorithms or software that are central to the research but not yet described in published literature, software must be made available to editors and reviewers. We strongly encourage code deposition in a community repository (e.g. GitHub). See the Nature Portfolio guidelines for submitting code & software for further information.

Data

Policy information about availability of data

All manuscripts must include a data availability statement. This statement should provide the following information, where applicable:

- Accession codes, unique identifiers, or web links for publicly available datasets
- A description of any restrictions on data availability
- For clinical datasets or third party data, please ensure that the statement adheres to our policy

The datasets generated during and/or analysed during the current study are available from the corresponding author on reasonable request.

Field-specific reporting

Please select the one below that is the best fit for your research. If you are not sure, read the appropriate sections before making your selection.

☒ Life sciences ☐ Behavioural & social sciences ☐ Ecological, evolutionary & environmental sciences

For a reference copy of the document with all sections, see nature.com/documents/nr-reporting-summary-flat.pdf

Life sciences study design

All studies must disclose on these points even when the disclosure is negative.

Sample size	No sample-size calculation was performed. We used for these experiments, MucilAir pool tissues (https://www.epithelix.com/products/mucilair). These tissues are reconstituted from a mixture of nasal polyp epithelial cells originating from 14 healthy donors. This highly standardized tissue culture model was developed to reduce inter-individual and inter-assay variability and was used in previous studies where we compared tissue response to respiratory infections, including by transcriptomic analysis.
Data exclusions	Individual cells with high number of reads mapping of mitochondrial genes, therefore damaged or dead cells, were excluded from the analysis. This is common practice in scRNA-seq studies.
Replication	For all experiments, other than scRNA-seq analysis, all attempts at replication were successful. For scRNA-seq analysis We used for these experiments, MucilAir pool tissues (https://www.epithelix.com/products/mucilair). These tissues are reconstituted from a mixture of nasal polyp epithelial cells originating from 14 healthy donors. This highly standardized tissue culture model was developed to reduce inter-individual and inter-assay variability and was used in previous studies where we compared tissue response to respiratory infections, including by transcriptomic analysis.
Randomization	Samples were allocated randomly
Blinding	Investigators were blinded to group allocation during data collection and analysis

Reporting for specific materials, systems and methods

We require information from authors about some types of materials, experimental systems and methods used in many studies. Here, indicate whether each material, system or method listed is relevant to your study. If you are not sure if a list item applies to your research, read the appropriate section before selecting a response.

Materials & experimental systems

n/a	Involved in the study	
☐	☒	Antibodies
☐	☒	Eukaryotic cell lines
☒	☐	Palaeontology and archaeology
☒	☐	Animals and other organisms
☒	☐	Human research participants
☒	☐	Clinical data
☒	☐	Dual use research of concern

Methods

n/a	Involved in the study	
☒	☐	ChIP-seq
☐	☒	Flow cytometry
☒	☐	MRI-based neuroimaging

Antibodies

Antibodies used	Mouse monoclonal anti-IVA Ab Chemicon®; Alexa Fluor 488 Invitrogen™
Validation	The primary mouse monoclonal anti-IVA was internally validated on positive control samples (cell lines infected withinfluenza A virus) before being used in our study

Eukaryotic cell lines

Policy information about cell lines

Cell line source(s)	We used Mucilair tissues, human airway epithelia purchased by Epithelix company in Geneva
Authentication	We used Mucilair tissues, human airway epithelia purchased by Epithelix company in Geneva
Mycoplasma contamination	Mucilair tissues were tested negative for mycoplasma contamination

Commonly misidentified lines
(See ICLAC register)

Name any commonly misidentified cell lines used in the study and provide a rationale for their use.

Flow Cytometry

Plots

Confirm that:

☒ The axis labels state the marker and fluorochrome used (e.g. CD4-FITC).

☒ The axis scales are clearly visible. Include numbers along axes only for bottom left plot of group (a 'group' is an analysis of identical markers).

☒ All plots are contour plots with outliers or pseudocolor plots.

☒ A numerical value for number of cells or percentage (with statistics) is provided.

Methodology

Sample preparation	Mucilair tissued were dissociated with triplE, permeabilized with Perm/Wash Buffer RUO (554723 BD Biosciences-US) and stained in order to detect intracellular influenza virus and therefore distinguish infected from uninfected cells
Instrument	A MoFlo Astrios Cell Sorter (Beckman Coulter) was used
Software	BD FACSDiva Software was used to collect and analysed flow cytometry data
Cell population abundance	Sorting was not performed on permeabilized stained cells, but on live cells Hoechst +/DRAQ7 –. This double staining allowed us to sort live cells (70-80% of the total population), distinguishing them from dead cells (Draq7 -) or debris (Hoechst -/ DRAQ7 –).
Gating strategy	The uninfected gating control was defined using uninfected cells stained with both the primary and the secondary antibodies.

☒ Tick this box to confirm that a figure exemplifying the gating strategy is provided in the Supplementary Information.
